# Incidence and Mortality Trends in German Women with Breast Cancer Using Age, Period and Cohort 1999 to 2008

**DOI:** 10.1371/journal.pone.0150723

**Published:** 2016-03-02

**Authors:** Shoma Berkemeyer, Dorothea Lemke, Hans Werner Hense

**Affiliations:** 1 Hochschule für Gesundheit, Department of Community Health, Bochum, Germany; 2 Institute of Epidemiology and Social Medicine, Medical Faculty, Westfälische Wilhelms-Universität Münster, Germany; 3 Epidemiological Cancer Registry North Rhine-Westphalia, Münster, Germany; The University of Hong Kong, CHINA

## Abstract

Longitudinal analysis investigates period (P), often as years. Additional scales of time are age (A) and birth cohort (C) Aim of our study was to use ecological APC analysis for women breast cancer incidence and mortality in Germany. Nation-wide new cases and deaths were obtained from Robert Koch Institute and female population from federal statistics, 1999–2008. Data was stratified into ten 5-years age-groups starting 20–24 years, ten birth cohorts starting 1939–43, and two calendar periods 1999–2003 and 2004–2008. Annual incidence and mortality were calculated: cases to 100,000 women per year. Data was analyzed using glm and apc packages of R. Breast cancer incidence and mortality increased with age. Secular rise in breast cancer incidence and decline in mortality was observed for period1999-2008. Breast cancer incidence and mortality declined with cohorts; cohorts 1950s showed highest incidence and mortality. Age-cohort best explained incidence and mortality followed by age-period-cohort with overall declining trends. Declining age-cohort mortality could be probable. Declining age-cohort incidence would require future biological explanations or rendered statistical artefact. Cohorts 1949–1958 could be unique in having highest incidence and mortality in recent time or future period associations could emerge relatively stronger to cohort to provide additional explanation of temporal change over cohorts.

## Introduction

Western nations’ age-standardized incidence and mortality rates of breast cancer were 90–110 and 15–22 per 100,000 women, respectively, for the year 2010 [1]. Germany in 2009–10 reported age-standardized breast cancer incidence and mortality rates of 120 and 24 per 100,000 women, respectively [2]. Declining breast cancer mortality rates in Western nations have been reported consistently in literature, such as, in the late nineties [3], early 2000s [4, 5], 2011 [6], and recently in 2014 [7]. Better survival has been attributed to improved management and treatment of women [3, 5], such as, advanced chemotherapy [5] and adjuvant therapy [5, 8, 9]. Concomitantly rising trends in breast cancer incidence [10–13], with predicted future increase [13], and policy suggestion of prescient development of healthcare to absorb these demands [11, 13] have been reported. A recent prediction study reported on constancy in crude and decline in age-adjusted breast cancer incidence using age-period-cohort (APC) modeling [11]. Thirty percent of all incident cancers in women were breast cancers as reported by the Surveillance, Epidemiology, and End Results (SEER) in 2000 [14]. Thirty one percent of all incident cancers in women were breast cancers as per recent German statistics [2]. Breast cancer is on the rise in most developing nations, that it will remain a world health concern [1, 10, 15, 16].

Data for cancer rely on repeated point measures on same population over time to allow ecological analysis. Longitudinal time trends are main-stay of epidemiological analysis [5, 6, 17]. Randomized trials remain unrealistic in breast cancer disease setting; hence well-performed ecological studies are recommended [18, 19]. Longitudinal period analysis, often conducted in yearly intervals, has, in addition to yearly period, additional scales of time, such as, age and birth cohort. Age is usually adjusted in longitudinal analysis [5, 6]. Birth cohort, which is derived from period and age, shows inherent covariance with the two other time scales. APC analysis overcomes this by introducing *a priori* constraints, with concomitant report of absolute measures [11, 13, 20, 21]. Description of population based breast cancer programs are fraught with data interpretation challenges [5, 6, 11, 13, 22], where accounting for age, period and cohort augment descriptive data analysis [20, 21]. Thus, our objective was to provide a multi-scalar description of incidence and mortality due to breast cancer in women from Germany for the longitudinal period 1999–2008.

## Materials and Methods

### Data

Country-wide breast cancer disease statistics, i.e., new cases of breast cancer and deaths due to breast cancer, were available for time-period 1999 to 2008 from Robert Koch Institute, Berlin, which is the central German institution for all disease statistics [23]. Country-wide data aggregated by Robert Koch Institute is characterized by high reliability; minimum ninety percent of the data are without any missing values as per quality assurance [2]. We chose lower age limit of the youngest age group of women at 20–24 years (y), i.e., data from 20 y onwards, and in intervals of five years were obtained. Data was then categorized into ten age-groups: 20–24, 25–29, …, 65+y for the period 1999 to 2008, comprising our age variable. We further categorized each age-group into two, five-year periods of observation 1999–2003 and 2004–2008, which yielded the two period-groups. We obtained per age-group and per period-group the birth cohort year, 1939–43,…,1984–1988, which yielded our cohort-groups. Age-group stratified female population was obtained from the German federal government statistical data-base DeStatis [24].

We calculated incidence as the number of new cases with breast cancer compared to that age-group specific population per 100,000 per year. Mortality rate was calculated as the number of deaths due to breast cancer compared to that age-group specific population per 100,000 per year. We collated data using MS Excel (Microsoft Excel 2010, Munich, Germany) and analyzed it using R, a language and environment for statistical computing (R Foundation for Statistical Computing, Vienna, Austria) using packages glm and apc. We interpreted statistical significance as a 2-sided *P* value ≤ 0.05, consistent with a type I error of 5%. This study analyzed data records publically available as secondary data, and thus rendered exempt from ethical clearance.

### Rationale and Analysis

APC analysis discerns three types of time varying phenomena: age, period and cohort on rates thereby augmenting understanding of time-varying elements. Our primary analysis was conducted using glm package of R. Regressions were run for outcomes incidence and mortality. We assumed the number of cases in an age-group at period time to be a Poisson distribution. Age-group indicated the differing risks associated with different age-groups. In APC analysis age variations are linked to physiological, biological and social processes of aging, which are unrelated to the time-period or the birth cohort to which the individual belongs. Our period-group indicated the change in rate associated with all age-groups simultaneously over time. These variations result from external factors that equally affect all age-groups at a particular calendar time ranging from environmental, social and economic factors, e.g., famines, crises, wars, etc. Birth cohort groups indicated cohort specific characteristics, such as, use of hormonal contraceptives or hormone replace treatment (HRT) or parity habits, etc. These variations result from the unique exposure of cohort as they move across in time, classically conceptualized in research as an interaction term. In APC analysis, the cohort term represents a factor indexing the sum of all exposures experienced by a cohort from birth. Thus, APC analysis treats time-specific variables, otherwise treated as confounders, mediators, moderators and interaction terms, as individual independent factors of age, period and cohort. As published (9, 10), we used APC model as the logarithm of expected rate

$$\text{ln}\left(\text{E}\left[{\text{r}}_{ij}\right]\right)=\mu +\alpha_{i}+\beta_{j}+\gamma_{k},$$ (1)

where, μ was the mean contribution, α_*i*_ the contribution of age-group, β_*j*_ the contribution of period *j*, γ_*k*_ is the contribution of *k*th birth cohort, and where, *i* = 1, …, A, *j* = 1, …, P, and *k* = 1,…, C. *Per se* solving for this outcome is possible. But, in that we estimated cohort from age and period, we generated a three-way table indirectly, where generally only a two-way would have been available. This comes with two problems. First is that birth cohorts have a sequence of overlapping intervals and, second, there is a direct linear dependence between the effects of age, period and cohort. This problem in estimation of model parameters can be solved by using individual records of cohort, instead of calculating it from age and period, thereby generating a true three-way table. While this solution in APC analysis is not without critic, e.g., underestimation of numbers of cases in younger among older cohorts, etc., there are additional practical issues to the problem. Population data, such as federal statistics, demographics, registry data, etc. are usually available as periodic, largely yearly, aggregates instead of individual records. Thus, in order to find a solution in APC analysis, usually an extra constraint, which is over and above the three time scales age, period and cohort, is used. This can be done by using a weighted average of the three two-factor model solutions, where more weight is attached to the best fitting two-factor model; best fitting defined by smallest residual mean deviance. This method too has limitations, e.g., lack of standard errors, period effect appearing to be negligible, etc. Thus, methods, which concentrate on estimating the parameters using local curvatures, are usually in use.

Thus, in our study regressions were run using, fitting for age, age and period, age and cohort, and age, period and cohort, with basis (B) splines, thereby allowing curvatures with continuity at knots. Even in curvatures method there can be great fluctuation, e.g., in cohort curvatures at oldest and/ or youngest cohorts. Thus, reporting absolute values in APC analysis is required to keep reference in focus and caution in interpretation [11, 13, 20, 21]. As generally the practice, for our study the best-fitting model was defined as one that minimized Akaike information criterion (AIC). Models with lower residual deviance had better goodness of fit indicated by lower AIC. Differences between models were tested using Chi-square test. Results for age-group 65+ would not be truly comparable with other age-groups, since it was not a five-year age interval, but we refrained from information loss in older populations and hence included the age-group in our analysis.

### Sensitivity

Primary analysis was conducted using glm modeling. This was confirmed using apc modeling of R package providing internal validation of results. Our assumption thereof was that though the numbers obtained by both methods would be different, the direction, order and relation of results obtained by using two different techniques would be comparable. Apc modeling uses the first birth cohort as reference for subsequent cohorts, rendering the fits amenable to further caution in interpretation. Apc modeling allows generation of trend diagrams, which require confirmation with absolute data trends. Reference to absolute values has been, in general, recommended strongly for APC modeling [20, 21].

## Results

**Fig 1:**
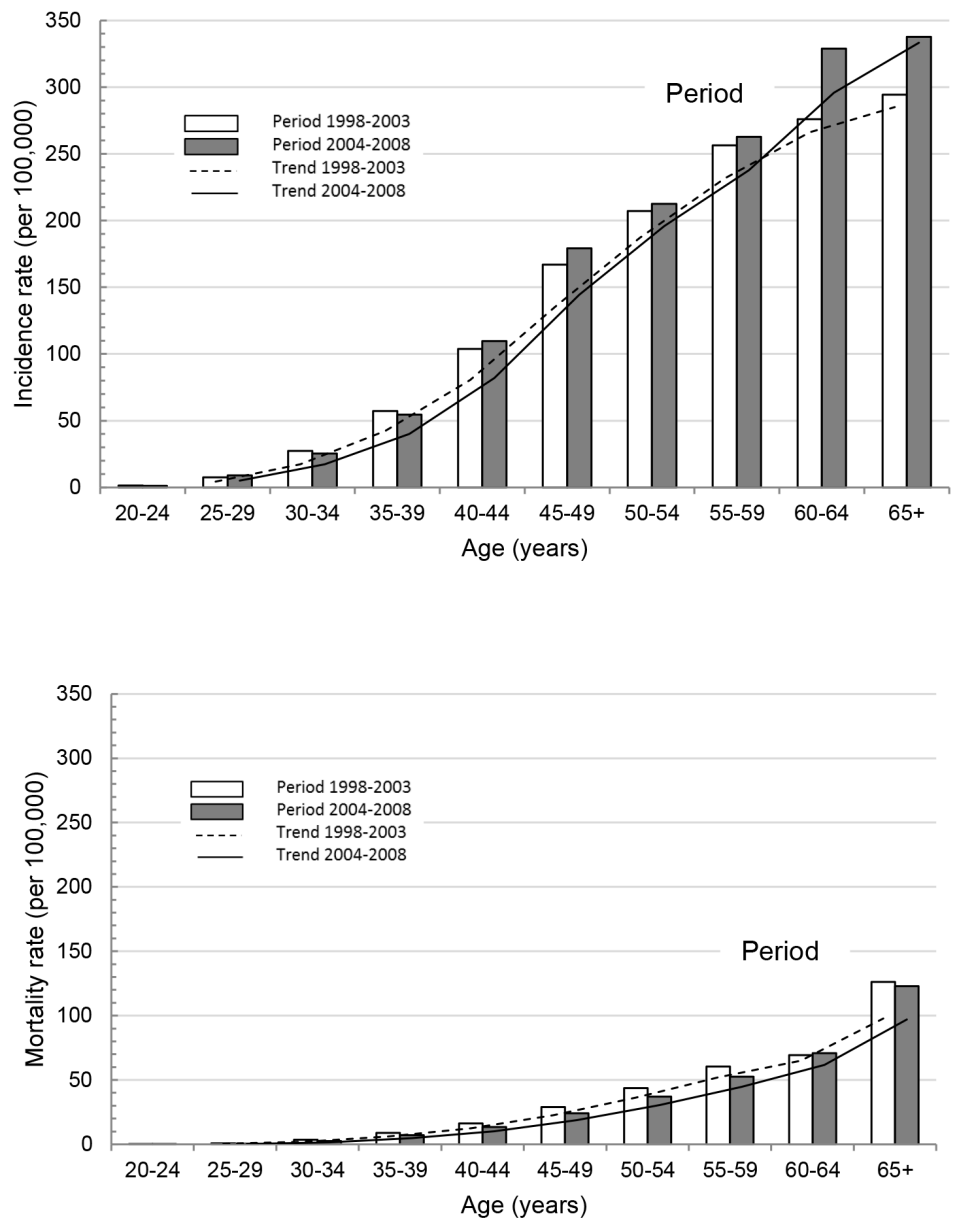
Breast Cancer (a) Incidence and (b) Mortality Across Age-Groups Over Two Time Periods 1993–2003 and 2004–2008.

Absolute data aggregates are reported in Table 1; we observed an increase in incidence over period (Fig 1A) and decrease in mortality over period (Fig 1B). We observed an increase in incidence over age (Fig 1A) and an increase in mortality over age (Fig 1B).

**Table 1:** Absolute Data Aggregates for Breast Cancer and Deaths due to Breast Cancer in German Women for Period 1999–2008.

No.	Age-group	Period-group	Cohort-group	NewCases (*n*)	Deaths (*n*)	Population (*n*)
1	20–24	1999–2003	1979–83	146	14	11,023,000
2	20–24	2004–2008	1984–88	151	7	11,776,000
3	25–29	1999–2003	1974–78	844	74	11,377,000
4	25–29	2004–2008	1979–83	1085	68	11,891,000
5	30–34	1999–2003	1969–73	3953	505	14,461,000
6	30–34	2004–2008	1974–78	3084	310	12,114,000
7	35–39	1999–2003	1964–68	9558	1465	16,691,000
8	35–39	2004–2008	1969–73	8527	1108	15,634,000
9	40–44	1999–2003	1959–63	16250	2530	15,647,000
10	40–44	2004–2008	1964–68	19255	2353	17,576,000
11	45–49	1999–2003	1954–58	24134	4172	14,463,000
12	45–49	2004–2008	1959–63	27966	3743	15,602,000
13	50–54	1999–2003	1949–53	27128	5718	13,103,000
14	50–54	2004–2008	1954–58	30573	5312	14,380,000
15	55–59	1999–2003	1944–48	32795	7748	12,797,000
16	55–59	2004–2008	1949–53	33492	6691	12,749,000
17	60–64	1999–2003	1939–43	41656	10458	15,092,000
18	60–64	2004–2008	1944–48	40214	8664	12,225,000
19	65+	1999–2003	1934–38	128810	55201	43,782,000
20	65+	2004–2008	1939–43	159487	58060	47,225,000

Glm modeling showed age-cohort (AC) and age-period-cohort (APC) fits to have the lowest AIC (Table 2). For incidence, APC fit had the lowest residual deviance but in negative range, suggesting that the model could have been over-fitted. Further, APC fit was not significantly different from AC fit, suggesting that the inclusion of the P term did not sufficiently improve the model. For mortality, AC fit had the lowest residual deviance with goodness of fit (AIC) similar to APC fit, suggesting AC fit could be the best fit for our study-specific cohort over time-period 1999–2008.

**Table 2:** Age-Period-Cohort Modeling for Incidence and Mortality of Breast Cancer in Women using glm of R package.

Outcome	Model*	Residual deviance	AIC**
Incidence			
	Age^a^^,^^b^^,^^c^	0.002	2382.80
	Age and period^a^^,^^d^^,^^e^	0.058	820.19
	Age and cohort^b^^,^^d^^,^^f^	0.00000007	261.25
	Age, period and cohort^c^^,^^e^^,^^f^	-0.00000000001	261.25
Mortality			
	Age^g^^,^^h^^,^^i^	0.033	536.11
	Age and period^g^^,^^j^^,^^k^	0.020	407.56
	Age and cohort^h^^,^^j^^,^^l^	0.000000000006	223.54
	Age, period and cohort^i^^,^^k^^,^^l^	0.000000000008	223.54

* Difference between models tested using Chi-square test of difference (models which do not differ or differ less are more similar to each other).

^a^ ≤ 0.001

^b^ ≤ 0.001

^c^ ≤ 0.001

^d^ ≤ 0.001

^e^ ≤ 0.001

^f^ ≤ not significant

^g^ ≤ 0.001

^h^ ≤ 0.001

^i^ ≤ 0.001

^j^ ≤ 0.001

^k^ ≤ 0.001

^l^ ≤ not significant

** AIC, Akaike information criterion (lower values indicate better goodness of fit)

Comparing the parameter estimates (β) for incidence, we observed that all categories of age and cohort were highly significant in AC fit. APC fit rendered younger birth cohorts with parameter estimates that were not significant. Concomitantly parameter estimate for period was highly significant (Table 3). Similar results were obtained for mortality (Table 3). AC fit for mortality showed all age and birth cohorts, with exception of 1944–48 birth cohort, to be highly significant in explaining mortality trends (Table 3). AC fits tended to have all estimates highly significant (Table 3) and with best goodness of fit (Table 2) for our study-specific cohort over time-period 1999–2008.

**Table 3:** Parameter Estimates Obtained (β) Using glm Modeling of Age-Cohort and Age-Period-Cohort for Incidence and Mortality of Breast Cancer.

Parameter	Incidence				Mortality			
	AC*		APC**		AC*		APC**	
	β***	Error	β***	Error	β***	Error	β***	Error
Age								
20–24 y	-11.81^a^	0.10	-11.32^c^	0.07	-12.24^c^	0.33	-14.13^c^	0.34
25–29 y	-9.88^a^	0.05	-9.44^a^	0.10	-10.73^a^	0.15	-12.41^c^	0.38
30–34 y	-8.65^a^	0.03	-8.26^a^	0.09	-9.36^a^	0.08	-10.83^a^	0.35
35–39 y	-7.95^a^	0.02	-7.63^a^	0.08	-8.66^a^	0.05	-9.91^a^	0.30
40–44 y	-7.31^a^	0.02	-7.03^a^	0.06	-8.23^a^	0.04	-9.28^a^	0.25
45–49 y	-6.76^a^	0.02	-6.54^a^	0.05	-7.84^a^	0.03	-8.68^a^	0.20
50–54 y	-6.52^a^	0.01	-6.35^a^	0.04	-7.59^a^	0.03	-8.22^a^	0.15
55–59 y	-6.28^a^	0.01	-6.17^a^	0.03	-7.41^c^	0.02	-7.83^a^	0.10
60–64 y	-6.03^a^	0.01	-5.98^a^	0.01	-7.25^a^	0.01	-7.46^a^	0.05
65+ y	-5.83^a^	0.003	-5.83^a^	0.003	-6.68^a^	0.004	-6.68^a^	0.004
Period	-	-	0.05^a^	0.01	-	-	-0.21^a^	0.05
Cohort								
1939–43	0.14^a^	0.004	0.08^a^	0.01	-0.03^a^	0.006	0.18^a^	0.05
1944–48	0.31^a^	0.008	0.20^a^	0.03	-0.003^d^	0.016	0.42^a^	0.10
1949–53	0.34^a^	0.01	0.17^a^	0.04	-0.15^a^	0.02	0.48^b^	0.15
1954–58	0.36^a^	0.01	0.15^b^	0.05	-0.31^a^	0.03	0.53^b^	0.20
1959–63	0.44^a^	0.02	0.16^c^	0.06	-0.50^a^	0.04	0.55^c^	0.25
1964–68	0.49^a^	0.01	0.16^c^	0.08	-0.69^a^	0.05	0.57^d^	0.30
1969–73	0.44^a^	0.02	0.06^d^	0.09	-0.90^a^	0.06	0.57^d^	0.35
1974–78	0.37^a^	0.03	-0.07^d^	0.10	-1.21^a^	0.09	0.46^d^	0.39
1979–83	0.58^a^	0.06	0.09^d^	0.10	-1.33^a^	0.19	0.55^d^	0.41
1984–88	0.54^a^	0.13	-	-	-2.10^a^	0.50	-	-

* AC, Age-cohort

** APC, Age-period-cohort

*** β, Parameter estimate and its significance

^a^ ≤ 0.001

^b^ ≤ 0.01

^c^ ≤ 0.05

^d^ not significant

Sensitivity analysis using Apc modeling yielded AC and age-period (AP) as significant models for both incidence and mortality (Table 4). AC had lowest residual deviance compared to AP model, suggesting AC to explain incidence and mortality better than AP. Residual deviance was same for AC and APC fits using Apc modeling. APC models though were not significant for our study-specific cohort in specific time period of 1999–2008.

**Table 4:** Age-Period-Cohort Modeling for Incidence and Mortality of Breast Cancer in Women using apc of R package—Sensitivity Analysis.

Outcome	Model*	Residual deviance	*p*-value
Incidence	Age-cohort	657.96	≤ 0.001
	Age-period-cohort	657.96	not significant
	Age-period	1009.31	≤ 0.001
Mortality	Age-cohort	315.03	≤ 0.001
	Age-period-cohort	315.03	not significant
	Age-period	476.73	≤ 0.001

* Default reference cohort 1939–43

**Fig 2:**
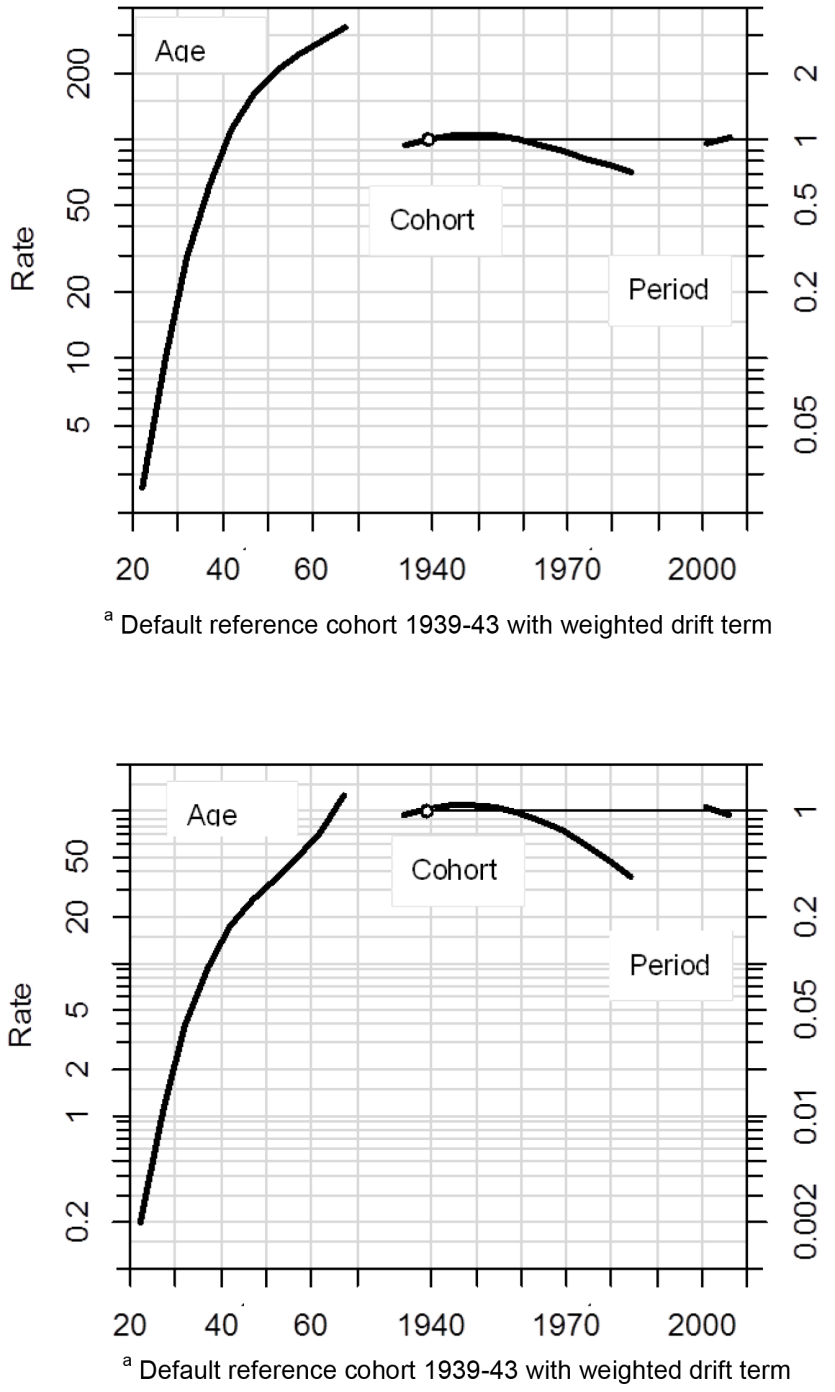
**Age-Period-Cohort Fitted Parameter Estimates (β) for (a) Incidence and (b) Mortality due to Breast Cancer.** Default reference cohort 1939–43 with weighted drift term.

Absolute values showed, that as period increased, incidence increased (Fig 1A) and mortality decreased (Fig 1B), and as age increased both incidence (Fig 1A) and mortality (Fig 1B) increased. Apc modeling obtained fitted values validated this absolute trend for incidence (Fig 2A) and mortality (Fig 2B) for age and period contributions, allowing our study to extrapolate for fitted cohort trends (Fig 2A and 2B). Cohort fits showed a decline in both incidence (Fig 2A) from cohort 1960 and decline in mortality (Fig 2B) from cohort 1950 onwards, where the observed decline in mortality was steeper than the decline in incidence.

## Discussion

We confirm secular decline in mortality of breast cancer in Germany from 1999 to 2008, which was best explained by age-cohort models followed by age-period-cohort models; concomitant absolute values also showed a decrease. Most ecological studies report secular decline in mortality of breast cancer over time period [3–7]. Our study additionally reports secular decline in incidence of breast cancer in Germany from 1999 to 2008 using age-cohort models; concomitant absolute values showed an increase. Most ecological studies report increasing incidence [10, 12, 13] of breast cancer over period time. Rapiti and colleagues using age-period-cohort modeling predicted decline in breast cancer incidence concomitant to increase in absolute numbers of breast cancers [11] over time. Yu and colleagues reported that age-cohort modeling best explained their data on breast cancer [13]. We confirm age-cohort to best explain our data, which was followed by age-period-cohort.

Our period-dependent results confirmed the worldwide trend of increasing incidence [10, 12, 13] and declining mortality [1, 4–7] of breast cancer with time. Our age-dependent incidence and mortality curves, both showing increase with age, were similar to 10-years older SEER data [4] and 20-years older U.S. data [15]. Our age-dependent mortality trends were somewhat also similar to Taiwanese data [22]. Age-dependent mortality and incidence of our study were different from trends observed for Asia-Oceania [15]. Greater uniformity in worldwide breast cancer mortality could be related to post-industrialization life-styles. Greater variance in worldwide breast cancer incidence could, likewise, be also related to absence of post-industrialization life-styles and/or genetic variations.

Our cohort-dependent results showed that 1949–53 had highest mortality and 1954–58 had highest incidence of breast cancer. That is, women in Germany born prior to 1950s, and those born after 1960s had apparently lower incidence and mortality of breast cancer compared to women born in the 1950s. The lower incidence and mortality in cohorts post 1960 were more pronounced than that of cohorts born prior to 1950s.Cohort results preceding 1950s can be possibly explained by war, the then standards of disease therapy and management, and cohort lifestyle practices, e.g., absence of HRT-use [4, 12, 25]. The declining incidence and mortality of cohorts born after 1960s poses a greater challenge. It could be possible that over time birth-cohort post-1960s are perhaps the healthiest of cohorts, as far as breast cancer is concerned. Secondary prevention strategies for breast cancer, justified by period-observed increasing morbidity [12, 13, 17] at period-based declining mortality [5, 6, 19], would then have begun in Germany in 1985 post this declining trend. Pertinent to this discussion is whether our study-observed declining incidence has a biological explanatory or is it a random observation.

Our study additionally reports period-dependent increasing incidence of breast cancer, well-confirmed by other studies [10, 12, 13], concomitant to age-cohort based declining incidence of breast cancer. Yu and colleagues using age-cohort modeling predicted breast cancer prevalence to rise sharply [13]. Prevalence of breast cancer would include the new cases of breast cancer with improved diagnostics [26] plus the running cases of breast cancer with longer survival [13], due to improved therapy and treatment [5, 8, 9]. Rapiti and colleagues using age-cohort modeling predicted decline in breast cancer incidence concomitant to absolute increase in total breast (prevalent) cancers [11].

Biological reasons such as deferred menopause with HRT-use in breast cancer etiology [12, 25] would be of lower to little relevance for our study’s birth cohorts born after 1960s; oldest till date would be 55 years old. Conflicting results have been reported for parity, age of first parity and age of menarche in breast cancer morbidity [27, 28]. Our study’s birth cohort post 1960s would be characterized by all these risk factors of lower parity, lower first age of parity and early menarche. Possibly, these factors are not as primary as perhaps obesity, a cohort characteristic of post-industrialization life-style [6, 25, 27, 28], in breast cancer morbidity. Simultaneous improvement in protective factors of primary prevention, such as, improvement in maternal and fetal health and health care services [29], as observed in other post-industrialized nations [26], could possibly attenuate risks partially. Contributions of risk factors of parity, age of first parity and age of menarche [27, 28] could be mediated by hormone-based contraceptives, a post-industrialization life-style characteristic. There have been rapid changes in composition of hormone-based contraceptives over time with a recent publication reporting inverse association between estrogen-use, component of hormonal contraceptives, and risk of young-onset breast cancer [11, 30].

Apart from biological explanations, which will require test of time, our observation of an age-cohort determined declining incidence could be as well a random observation. Our study’s older birth-cohorts would be longer in the analysis compared to younger cohorts. This association, dependent on age, would confound the present cohort contribution. The younger cohorts would require having the same chance of being as long in the analysis compared to older cohorts, necessitating a passage of another 40 to 60 years. Given this possibility, the currently observed best-fitting age-cohort dependent declining incidence of breast cancer could in future look different or be even obliterated. Harper [31] recently explained that reductions in major risk factors can be achieved both through cohort and period contributions, e.g., our study found both age-cohort and age-period-cohort to explain breast cancer outcome. An amount of uniformity of temporal change over multiple cohorts would exist parallel to specific cohort contributions, both which would complement each other in disease outcomes. Future research on this, thus, remains very pertinent.

The strength of our study is the use of three scales of time; age, period and birth cohort, which allowed a comprehensive analysis of incidence and mortality of breast cancer in Germany for the period 1999–2008. Our study observed period-based increasing incidence [13] can be, thus, amenable with cohort-based declining incidence as well as period-based declining mortality [11] with cohort-based declining mortality, which is a unique strength of APC analysis and our study’s. Our study is limited by classical ecological analysis, i.e., it being a trend analysis excludes causal inferences. We can, at best, only suggest explanations of the trends observed. Individual records would be inherently more reliable than our aggregates based analysis. Correspondingly, we observed these on a large, national population and refrained from any predictions and policy suggestions [20, 21], with our aim being to provide a multi-scalar view on morbidity and mortality of breast cancer with in-depth descriptive analysis.

Our study reports secular increase in incidence and decline in mortality of breast cancer for 1999 to 2008 using period analysis. Our study confirms increase in both incidence and mortality with age. Birth cohort analysis showed incidence and mortality to be decreasing after the decade of 1950s. Our study reports comprehensively on conflicting observations of time using age, period and cohort, and their relative strength to each other in explaining disease outcome, which might otherwise confound results. Our results were best explained by age-cohort models followed by age-period-cohort models, yielding deeper understanding of morbidity and mortality of breast cancer, a novelty of APC-analysis, and hence of our study.

## Conclusion

We conclude that while decline in age-cohort dependent mortality could be probable, decline in age-cohort dependent incidence would require either future biological explanations or rendered statistical artefact. Thus depending, cohorts 1949–1958 could be unique in having or not having highest incidence and mortality in recent time or future period contributions could emerge relatively stronger to cohort to provide additional explanation of temporal change over cohorts.

## Abbreviations

AIC: Akaike information criterion
APC: age-period-cohort method
Apc: age-period-cohort models of R Apc-modeling package
Glm: age-period-cohort models of R Glm modeling package
HRT: Hormone replacement treatment
SEER: Surveillance, Epidemiology, and End Results
